# Occipital Magnocellular VEP Non-linearities Show a Short Latency Interaction Between Contrast and Facial Emotion

**DOI:** 10.3389/fnhum.2020.00268

**Published:** 2020-07-09

**Authors:** Eveline Mu, David Crewther

**Affiliations:** Centre for Human Psychopharmacology, Swinburne University of Technology, Hawthorn, VIC, Australia

**Keywords:** magnocellular, non-linear VEP, emotion, contrast, V1

## Abstract

The magnocellular system has been implicated in the rapid processing of facial emotions, such as fear. Of the various anatomical possibilities, the retino-colliculo-pulvinar route to the amygdala is currently favored. However, it is not clear whether and when amygdala arousal activates the primary visual cortex (V1). Non-linear visual evoked potentials provide a well-accepted technique for examining temporal processing in the magnocellular and parvocellular pathways in the visual cortex. Here, we investigated the relationship between facial emotion processing and the separable magnocellular (K2.1) and parvocellular (K2.2) components of the second-order non-linear multifocal visual evoked potential responses recorded from the occipital scalp (O_Z_). Stimuli comprised pseudorandom brightening/darkening of fearful, happy, neutral faces (or no face) with surround patches decorrelated from the central face-bearing patch. For the central patch, the spatial contrast of the faces was 30% while the modulation of the per-pixel brightening/darkening was uniformly 10% or 70%. From 14 neurotypical young adults, we found a significant interaction between emotion and contrast in the magnocellularly driven K2.1 peak amplitudes, with greater K2.1 amplitudes for fearful (vs. happy) faces at 70% temporal contrast condition. Taken together, our findings suggest that facial emotional information is present in early V1 processing as conveyed by the M pathway, and more activated for fearful as opposed to happy and neutral faces. An explanation is offered in terms of the contest between feedback and response gain modulation models.

## Introduction

The magnocellular (M) visual system has been implicated in rapidly processing salient facial emotions, such as fear because it provides the main neural drive into the rapid collico-pulvinar route to the amygdala (Morris et al., 2001; Vuilleumier et al., 2003; de Gelder et al., 2011; Rafal et al., 2015; Méndez-Bértolo et al., 2016). The M pathway is a rapidly conducting neural stream providing motion and spatial localization information, as well as transient attention (Laycock et al., 2008). It possesses high gain for luminance contrast, and relative to the parvocellular (P) pathway it shows greater capability for high temporal and low spatial frequency stimulation. The P visual system processes in parallel to the M system, however it is less sensitive to luminance contrast, is chromatically (R/G) sensitive, and has a preference for low temporal and high spatial frequency stimulation. The P system is also considered to have slower conduction and it appears to not contribute directly to the collicular pathway (Livingstone and Hubel, 1988; Merigan and Maunsell, 1993).

Human anatomical evidence for the subcortical “low road” route (LeDoux, 1996) for emotional processing derives from functional magnetic resonance imaging (fMRI; Morris et al., 2001; Vuilleumier et al., 2003; Sabatinelli et al., 2009; de Gelder et al., 2011; Kleinhans et al., 2011; Rafal et al., 2015; Méndez-Bértolo et al., 2016), diffusion imaging (Tamietto et al., 2012; Rafal et al., 2015), magnetoencephalography (MEG; McFadyen et al., 2017) and computational modeling (Rudrauf et al., 2008; Garvert et al., 2014). Supporting the notion of rapid subcortical input to the amygdala, studies have found the estimated synaptic integration time for the subcortical pathway (80–90 ms) to be faster than that of the cortical visual pathway (145–170 ms; Morris et al., 1999; Öhman, 2005; Garvert et al., 2014; Silverstein and Ingvar, 2015; McFadyen et al., 2017). Furthermore, the superior colliculus comprises predominantly M neural inputs (Leventhal et al., 1985; Burr et al., 1994; Márkus et al., 2009).

Recently, these findings were confirmed electrocorticographically, where M-biased low spatial frequency fearful faces were found to evoke early activity in the lateral amygdala, 75 ms post-stimulus onset (Méndez-Bértolo et al., 2016). Additionally, several studies have reported faster and greater P100 amplitude responses to low spatial frequency fearful faces compared to neutral (Pourtois et al., 2005; Vlamings et al., 2009), with a recent study by Burt et al. (2017) pointing to specific M contribution. Taken together, the rapid colliculo-pulvinar-amygdala pathway forms the dominant hypothesis for the early facilitation of salient visual information processing (Öhman, 2005).

Critically, however, many of these studies only focus on how the salient visual information reaches the amygdala, and not what happens after. There is considerable evidence suggesting a relationship, or re-entry, between activity in the amygdala and primary visual cortex (V1; Morris et al., 1998; Sabatinelli et al., 2009) *via* the M pathway. The separation of M and P projections remains intact from retinal ganglion cells to V1 (Nassi and Callaway, 2009), with the M pathway terminating primarily in layer 4Cα of V1 and the P pathway terminating primarily in layer 4Cα of V1 (Fitzpatrick et al., 1985). However, little is known as to whether facial emotional stimuli reach V1 *via* M or P inputs, or with what timing. Also, direct inputs from the geniculo-cortical stream possess small receptive fields insufficient to code for a whole face. Hence, inputs to the occipital cortex from other regions that can code faces and particularly facial emotion are required.

It is possible to discriminate temporal M and P contributions to V1 with nonlinear multifocal visual evoked potentials (VEP; Baseler and Sutter, 1997; Klistorner et al., 1997; Jackson et al., 2013; Hugrass et al., 2018). In multifocal VEP experiments, multiple patches of light are flashed and de-correlated in pseudorandom binary sequences. Not only does this method allow for simultaneous recordings across the visual field, but it also analyses higher-order temporal nonlinearities through Wiener kernel decomposition (Sutter and Tran, 1992). The K1 kernel response measures the overall impulse response function of the neural system. The K2.1 response measures the nonlinearity (neural recovery) over one video frame, while K2.2 measures the recovery over two video frames (Sutter, 2000). Klistorner et al. (1997) proposed that the K2.1 response reflects M pathway activity due to its high contrast gain and a saturating contrast response function. Similarly, the main component (N95-P130) of the K2.2 response is thought to reflect P functioning as the response waveform has low contrast gain and a non-saturating contrast response function (Klistorner et al., 1997). However, the notion of isolating M and P contributions to cortical processing has been questioned, with Skottun (2013) suggesting that the M signal cannot be isolated by high temporal frequencies because temporal filtering occurs between the lateral geniculate nucleus and V1, with a reduction in temporal frequency cutoff of around 10 Hz found in primate single-cell studies (Hawken et al., 1996). Further, Skottun (2014) proposed that attributing VEP responses to the M and P systems based on contrast-response properties is problematic because of the mixing of inputs. In response, we argue that non-zero higher-order Wiener kernels of the VEP exist precisely because of such cortical filtering. Thus, the M and P nonlinear contributions to the VEP are heavily weighted to the first and second slices of the second-order response respectively (Klistorner et al., 1997; Jackson et al., 2013), based on contrast gain, contrast response functions, and peak latencies, and hence are easily separable. This identification has been backed up by recent studies investigating individual differences in behavior and physiology with correlations demonstrated between psychophysical flicker fusion frequencies and K2.1 peak amplitudes from the multifocal VEP (Brown et al., 2018). Here, we address the question of whether different emotional states affect the nonlinear structure of occipitally generated evoked responses. Any variation in response to emotional salience likely relates to the functional connections from emotion parsing regions such as the amygdala to the visual cortex.

The question of whether facial emotional stimuli reach V1 *via* M or P inputs has not been reported in human non-linear multifocal VEP recordings. Thus, the current study aimed to utilize this well-validated technique to evaluate whether emotional stimuli such as fearful, happy, and neutral faces would affect the early cortical (V1) M and P signatures.

## Materials and Methods

### Participants

Fourteen participants (nine males, fix females; *M* = 24 years, *SD* = 3.65 years) gave written informed consent and participated in the experiment at the Swinburne University of Technology, Melbourne, Australia. The first author was included in the sample. All participants had normal, or corrected-to-normal, visual acuity, and no neurological condition. The study was conducted with the approval of the Swinburne Human Research Ethics Committee and following the code of ethics of the Declaration of Helsinki.

### Visual Stimuli

**Figure 1:**
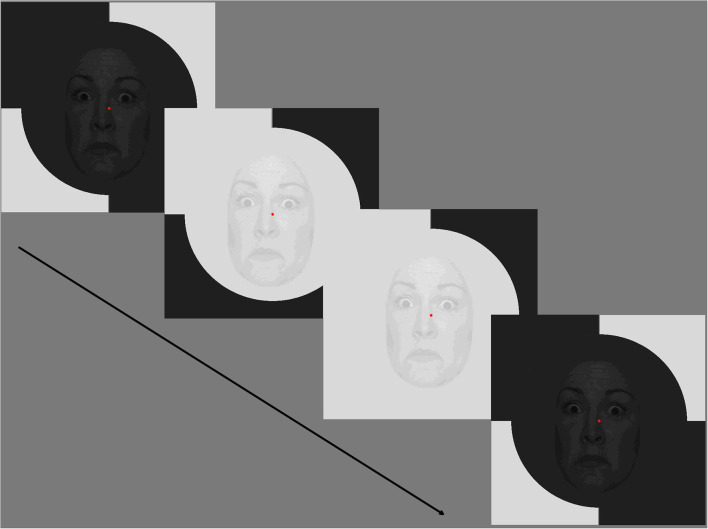
Example of a fearful condition with 70% temporal modulation. Stimuli comprised of pseudorandom brightening/darkening of fearful, happy, neutral faces (or no face) with surround patches decorrelated from the central face-bearing patch. For the central patch, the spatial contrast of the faces was 30% while the temporal contrast of the per-pixel luminance increment/decrement was 10% or 70%. Note that for each condition (happy, fearful, neutral) faces of different actors changed every second, but maintained emotional state. Consent was obtained for the use of NimStim stimuli.

The achromatic stimuli were presented on a 60 Hz LCD monitor (ViewSonic) with linearised color output (measured with a ColorCal II), at a viewing distance of 70 cm. The 9-patch multifocal dartboard was created using VPixx software (version 3.21)^1^, with a 5.4° diameter central patch and two outer rings of four patches (21.2° and 48° diameter; Hugrass et al., 2018). The luminance for each patch fluctuated between two levels, under the control of a pseudorandom binary m-sequence (*m* = 14) and modulated at the video frame rate of 60 Hz. All participants completed eight VEPs of varying temporal luminance contrasts (10% and 70% Michelson) for the outer patches, with an overall mean screen luminance of 65cd/m^2^. Of important note, unlike previous multifocal VEP studies (Sutherland and Crewther, 2010; Jackson et al., 2013; Crewther et al., 2015, 2016; Burt et al., 2017; Hugrass et al., 2018) that used a diffuse central patch, fearful, happy, neutral faces (or no face) from the Nimstim Face Set (Tottenham et al., 2009) were superimposed on the luminance fluctuation of the central patch. The spatial contrast (Michelson) of the central patch was either 30% (face) or 0% (no face). Thus, each pixel of this central image underwent a pseudorandom binary sequence of increases and decreases in luminance (Figure 1).

Stimuli comprised pseudorandom brightening/darkening of fearful, happy, neutral faces (or no face) with surround patches decorrelated from the central face-bearing patch. For the central patch the spatial contrast of the faces was 30% while the temporal contrast of the per-pixel brightening/darkening was 10% or 70% (Klistorner et al., 1997; Jackson et al., 2013; Brown et al., 2018; Hugrass et al., 2018).

M-sequences allow information from all stimulus patches to be available through rotation of the starting point of the binary sequence for each patch, resulting in full decorrelation (Sutter, 2000). For this experiment, we only analyzed responses to the central patch. Separate recordings were made with happy, neutral, fearful, and no face conditions at the different temporal contrasts. For each experimental condition, the m-sequences were split into four approximately one-minute recording segments, with the recordings lasting 32 min in total for the eight conditions. Participants were instructed to maintain strict fixation on the central patch during the recordings and to rest their eyes between recordings.

### Non-linear VEP Recording and Analysis

Non-linear achromatic multifocal VEPs were recorded using a 64-channel Quickcap and Scan 4.5 acquisition software (Neuroscan, Compumedics). Electrode site Fz served as ground and linked mastoid electrodes were used as a reference (Burt et al., 2017; Hugrass et al., 2018). EOG was monitored by positioning electrodes above and below the left eye.

EEG data were processed using Brainstorm (Tadel et al., 2011). EEG data were band-pass filtered (0.1–40 Hz) and signal space projection was applied to remove the eye-blink artifact. Custom Matlab/Brainstorm scripts were written for the multifocal VEP analyses to extract K1, K2.1, and K2.2 kernel responses for the central patch. K1 is the difference between responses to the light and dark patches. K2.1 measures neural recovery over one frame by comparing responses when a transition did or did not occur. Similarly, K2.2 measures neural recovery over two frames but includes an interleaving frame of either polarity (refer to Klistorner et al., 1997; Sutter, 2000 for in-depth descriptions of the kernels).

For each participant, the electrode with the highest amplitude responses was selected for group-level averages. The highest amplitude responses were recorded at Oz for all participants. Peak amplitudes and latencies of kernels K1, K2.1 and K2.2 were identified using Igor Pro 8.03 (Wavemetrics, Lake Oswego), establishing latency windows for peak identification from the grand mean averages. Values were then exported to SPSS (Version 20, IBM). To control for amplitude outliers a Winsorizing approach (Hastings et al., 1947; Dixon, 1960) was applied, limiting extreme values to the values of the 95th and 5th percentiles. For this outlier control, the data for the eight conditions associated with K2.1_N60-P90_ (FE70%: 2 cases; HA10%:1) and K2.1_N103-P127_ (FE70%: 1 case; HA70%: 2 cases; HA10%: 1 case; NE70: 1 case) amplitudes were adjusted for a small number of cases. These values were then used for linear mixed-effect modeling analysis and to present the mean values shown in the figures below. To allow for multiple comparisons, an alpha value of 0.006 was used for any follow-up pairwise comparisons (based on the eight stimulus conditions: FE30%, HA30%, NE30%, NoForm30%, FE70%, HA70%, NE70%, NoForm70%), and a 99% confidence interval was used for comparisons of marginal means associated with significant interactions.

## Results

**Figure 2:**
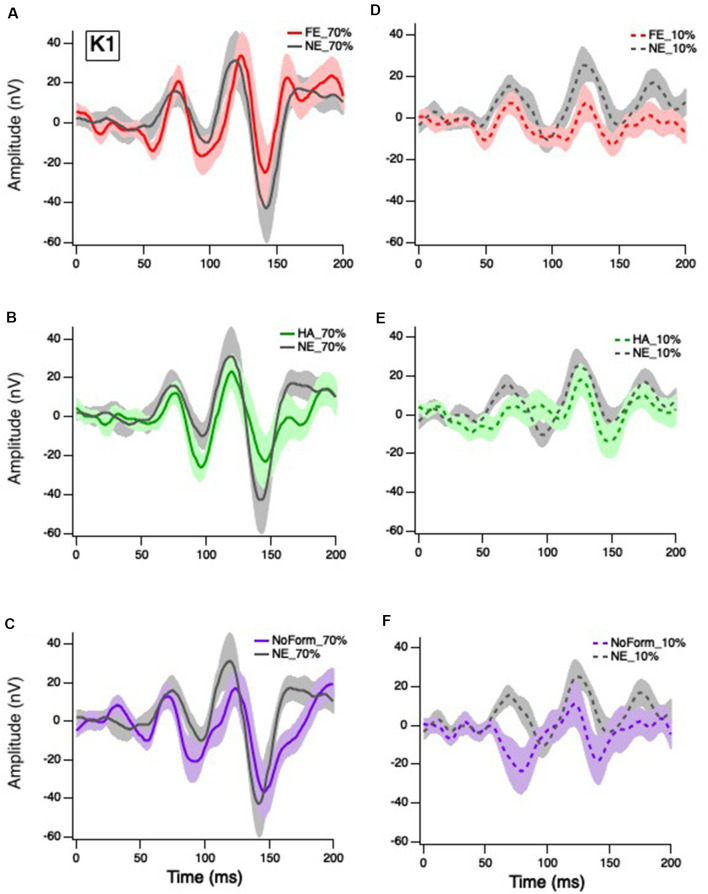
Grand mean average K1 responses. Solid red, green and purple lines correspond to the averaged waveforms for the 70% temporal contrast conditions with **(A)** fearful, **(B)** happy, and **(C)** no form stimuli superimposed in the central stimuli, respectively, all plotted against neutral (gray). Dashed red, green and purple lines correspond to the averaged waveforms for the 10% temporal contrast conditions with **(D)** fearful, **(E)** happy, and **(F)** no form superimposed in the central patch, respectively, all plotted against (gray).

**Figure 3:**
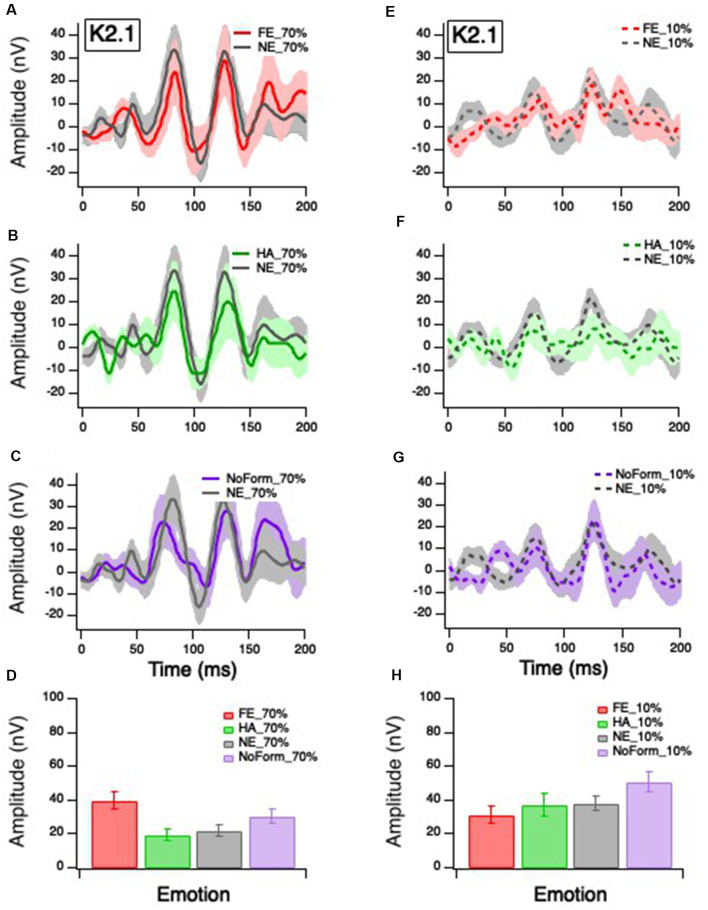
Grand mean average K2.1 responses. Solid red, green and purple lines correspond to the averaged waveforms for the 70% temporal contrast conditions with **(A)** fearful, **(B)** happy, and **(C)** no form stimuli superimposed in the central stimuli, respectively, all plotted against (gray). Dashed red, green and purple lines correspond to the averaged waveforms for the 10% temporal contrast conditions with **(D)** fearful, **(E)** happy, and **(F)** no form superimposed in the central patch, respectively, all plotted against (gray). Mean peak amplitude values of K2.1N60-P90 for 70% and 10% temporal contrast conditions across all emotions are shown in **(G,H)**, respectively, to illustrate the significant emotion by contrast interaction.

**Figure 4:**
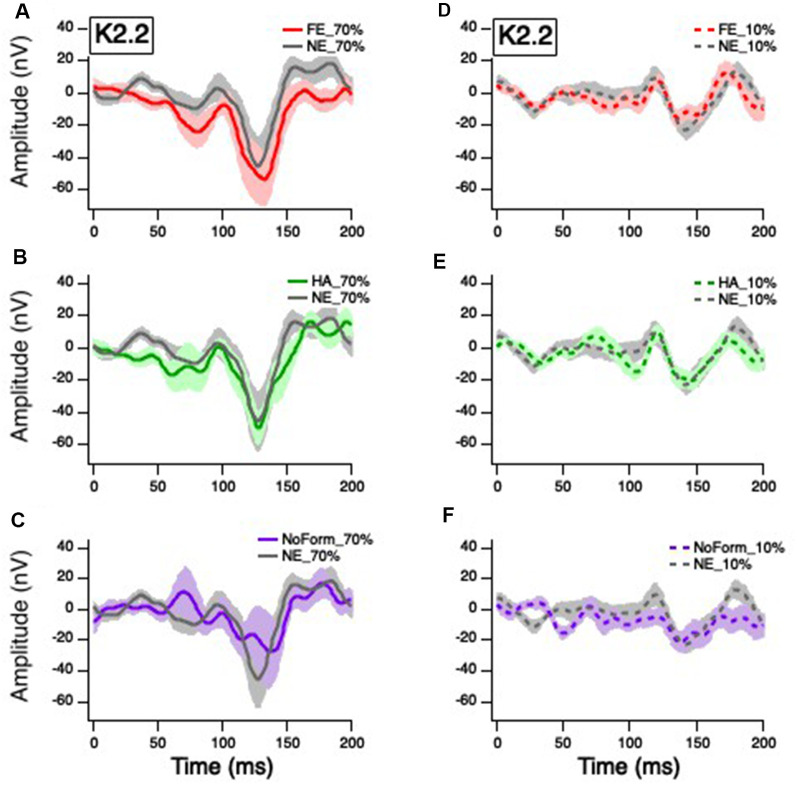
Grand mean average K2.2 responses. Solid red, green, gray, and purple lines correspond to the averaged waveforms for the 70% temporal contrast conditions with **(A)** fearful, **(B)** happy, neutral, and **(C)** no form stimuli superimposed on the central patch, respectively. Dashed red, green, gray, and purple lines correspond to the averaged waveforms for the 10% temporal contrast conditions with **(D)** fearful, **(E)** happy, neutral stimuli, and **(F)** no form superimposed on the central patch, respectively.

Grand averages for the K1, K2.1, and K2.2 responses were calculated for all experimental conditions (happy, fearful and neutral facial expressions, low and high temporal contrasts) and are presented in Figures 2–4, respectively. As expected, the cortically recorded VEP responses produced variations in amplitude according to contrast across all kernels (Klistorner et al., 1997). Separate linear mixed-effects models were computed to investigate the effects of emotion (fear, happy, neutral, no form) and temporal contrast (10%, 70%) on separate early and late peak amplitudes of the K1 (N58-P80; N94-P118), K2.1 (N60-P90; N103-P127), and K2.2 (N85-P104; N119-P157) responses. Time windows for peak estimation were established to account for individual differences across conditions. Some departures from the data of Klistorner et al. (1997), Jackson et al. (2013), and Hugrass et al. (2018) are apparent, due to differences in stimulus frame rate, reference/ground location (mastoid/Fz vs. Fz/mastoid).

### K1 Amplitude

Klistorner et al. (1997) suggested that the first-order response (K1) is produced by complex interactions between the M and P pathways. Separate linear-mixed model analyses for early and late K1 peak-trough amplitudes produced no significant main effects of emotion, K1_N58-P80_: *F*_(3,27)_ = 1.202, *p* = 0.328; K1_N94-P118_: *F*_(3,27)_ = 0.748, *p* = 0.535; nor were there any significant emotion by contrast interactions, K1_N58-P80_: *F*_(2,53)_ = 0.139, *p* = 0.870; K1_N94-P118_: *F*_(2.55)_ = 0.444, *p* = 0.644. As expected, there was a significant main effect of contrast on K1 but only for the earlier peak amplitudes, with greater responses at 70% (Figures 2A–C) than 10% temporal contrast (Figures 2D–F), K1_N58-P80_: *F*_(1,62)_ = 7.895, *p* = 0.007. In summary, short-latency K1 peak amplitudes are greater in magnitude when the central patch is modulated at high contrast, but they are not affected by facial emotion.

### K2.1 Amplitude

Klistorner et al. (1997) and Jackson et al. (2013) suggest that the K2.1_N60-P90_ waveform is of M pathway origin, based on contrast gain, contrast saturation, and peak latencies. Figure 3 illustrates K2.1 waveform for 70% temporal contrast (Figures 3A–C) and 10% temporal contrast (Figures 3D–F). One can see that the mean value of no form 10% in Figure 3H appears larger than the other emotions, which may suggest that the inclusion of facial stimuli in the central stimulus patch appears to have had some effect.

The linear-mixed model analysis showed a significant main effect of contrast on K2.1_N60-P90_ amplitude, *F*_(1,85)_ = 10.688, *p* = 0.002, but no significant main effect of emotion, *F*_(3,46)_ = 2.26, *p* = 0.094. There was a significant interaction between emotion and contrast, *F*_(3,41)_ = 4.823, *p* = 0.030, with the greatest amplitude for fearful faces in the 70% temporal contrast (Figure 3D), and greatest amplitude for no form in the 10% temporal contrast condition (Figure 3H). To ensure that the no form condition did not induce spurious effects, we conducted a *post hoc* separate linear mixed effect model without the no form condition and found a significant main effect of contrast, *F*_(1,63)_ = 5.399, *p* = 0.023, and significant emotion and contrast interaction, *F*_(2,52)_ = 4.951, *p* = 0.011.

No significant main effects or interactions were found for the later K2.1 peaks (K2.1_N103-P127_: *p* > 0.05).

### K2.2 Amplitude

Previous studies (Jackson et al., 2013) indicate that the small early K2.2_N85-P104_ peak is also of M origin. The linear mixed-effect model showed there was no significant main effect of contrast on the K2.2_ N85-P104_ amplitude, *F*_(1,48)_ = 1.025, *p* = 0.316. There was, however, a significant main effect of emotion, *F*_(3,41)_ = 7.012, *p* = 0.001, with greater amplitude for the no form condition compared to happy (*M*_diff_ = −20.876, *p* = 0.002) and neutral (*M*_diff_ = −20.290, *p* = 0.004) faces. There was no significant emotion by contrast interaction, *F*_(3,41)_ = 1.813, *p* = 0.160.

The second peak K2.2_N119-P157_ is thought to be of P origin (Jackson et al., 2013). Figure 4 illustrates a greater K2.2_N119-P157_ amplitude to 70% temporal contrast (Figures 4A–C) compared to 10% temporal contrast (Figures 4E–G), compared to K2.1 (Figure 3). As such, the linear mixed-effect model produced a significant main effect of contrast, *F*_(1,66)_ = 40.251, *p* < 0.001. There was no significant main effect of emotion, *F*_(3,39)_ = 0.109, *p* = 0.954, or interaction between contrast and emotion, *F*_(3,39)_ = 0.015, *p* = 0.997. Overall, it suggests that any emotional effect on the occipital VEP is of M and not P origin.

## Discussion

Nonlinear multifocal VEP recordings of the visual cortex have become perhaps the best available method for measuring human M and P temporal processing (Baseler and Sutter, 1997; Klistorner et al., 1997; Jackson et al., 2013; Brown et al., 2018; Hugrass et al., 2018). These studies typically examine M and P responses to flashing unstructured patches with a range of temporal contrasts, although Baseler and Sutter (1997) used contrast reversing checkerboards. However, no study to date has extended this technique to controlled luminance fluctuation of emotional faces, where, despite the random flicker, a clear percept of facial emotion is possible.

Considering the M and P pathways are known to contrast saturating and non-saturating, respectively (Kaplan et al., 1990; Klistorner et al., 1997; Jackson et al., 2013), there was no surprise that we found overall minimal K2.1 response differences between 10% and 70% temporal contrast, but greater difference when compared to K2.2_N119-P157_ waveforms. While some divergence in overall appearance of kernel waveforms compared with previous publications was observed, this can be partly explained by electrical reference/ground choices (aural medulla ref/Fz ground) rather than Fz as a reference with the aural ground as used by Klistorner et al. (1997) and Jackson et al. (2013). Another possible explanation for variation in response amplitudes relates to the presence or not of a facial percept. The presence of a percept implies higher-order visual processing that may result in feedback in area V1 (Fang et al., 2008). Also, the facial stimuli are likely to activate orientation-selective receptive fields of neurons in area V1 which the no form stimuli are less likely to stimulate, with differences in latency and waveform (Crewther and Crewther, 2010).

Based on the popular notion that the M pathway feeds into the colliculo-pulvinar-amygdala for rapid emotional processing we were interested in whether emotional content would have any effect on early occipital kernel responses. Interestingly, at the 70% temporal contrast level, we found fearful faces produced greater K2.1 amplitude compared to happy faces (which produced the smallest K2.1 amplitude) and neutral faces, which aligns with previous measures showing stronger and faster amygdala activation to fearful *cf* neutral faces (Öhman, 2005; Adolphs, 2008; Garvert et al., 2014; Méndez-Bértolo et al., 2016) and early visual cortical ERP by emotional faces (Vlamings et al., 2009; Burt et al., 2017). Before the current study, little was known as to the functional anatomy by which facial emotional information reaches V1, and with what timing. Thus, the current study provides evidence that emotional information is included in the first evoked response recording in V1 and is conveyed through the M pathway. Also, the recent literature on the normalization model of attention (Reynolds and Heeger, 2009; Herrmann et al., 2010; Zhang et al., 2016) needs to be considered, wherein neuronal firing rates of cortical neurons are dependent on the extent of the attentional field. Specifically, it has been found that both negative and positive emotional faces increase V1 activity relative to neutral faces, but at the same time, negative emotions narrow the attention field in V1 while positive emotion broadens the attention field (Zhang et al., 2016). Such articles introduce the notion of response gain as an attentional effect.

Emotional salience acts similarly to attention, with neural theories invoking response gain modulation of the pulvinar by amygdalar activity (Williams et al., 2004; van den Bulk et al., 2014). Previous studies have found the pulvinar to be crucial in gating and controlling information outflow from V1 (Purushothaman et al., 2012). Some studies (Vlamings et al., 2009; Attar et al., 2010; Burt et al., 2017) have found contrast response gain effects of the amygdala to fearful expressions to increase hMT and extrastriate early cortical responses (i.e., P100), thus potentially explaining why the M component, which should be saturated at 70% contrast, is being altered by emotional expression. Moreover, primate data are supportive, showing fast conducting projections from the inferior pulvinar to area middle temporal (MT; Warner et al., 2010; Kwan et al., 2019). But, while there is evidence of strong pulvinar-amygdala input, there is little evidence of a direct amygdala-pulvinar feedback pathway. The absence of such a pathway presents a problem in explaining very rapid changes in visual processing. However, transmission modulation of the pulvinar by the amygdala through verified projections onto the Thalamic Reticular Nucleus (TRN; Zikopoulos and Barbas, 2012), acting as an “emotional attention” mechanism (John et al., 2016), is highly plausible. This idea is further strengthened with evidence from optogenetic manipulation of amygdala activity producing strong contrast gain effects (Aizenberg et al., 2019).

Cortico-cortical feedback of emotional parsing by the amygdala back to the visual cortex is an alternative mechanism demanding exploration. The amygdala possesses myriad connections with the extrastriate cortex, including the insular cortex (Jenkins et al., 2017). Another alternative feedback pathway relates to the orbitofrontal cortex (OFC), a recipient of amygdala projections feeding information back to V1, with a role in further evaluation of the salient information. Kveraga et al. (2007) reported M information projected rapidly and early (~130 ms) to the OFC. Furthermore, analyses of effective connectivity using dynamic causal modeling showed that M-biased stimuli significantly activated pathways from the occipital visual cortex to OFC (Kveraga et al., 2007). However, these multisynaptic pathways likely have slower conduction to the striate cortex, and hence are less likely to contribute to the early K2.1 VEP component.

The biological and social significance of the human face, as a shape, needs to also be considered when interpreting our results. Previous studies have reported faces to capture attention more efficiently than non-face stimuli (Theeuwes and der Stigchel, 2006; Langton et al., 2008; Devue et al., 2009). For example, Langton et al. (2008) found that participants’ ability to search an array of objects for a target butterfly was slowed when an irrelevant face appeared in the array. This demonstrates that even when a non-face object is the target of a goal-directed search, the presence of a face prevails over other stimuli. However, electrophysiologically, Thierry et al. (2007) found that when showing pictures of faces and cars, it was not the category that evoked a greater N170 amplitude, but rather the within-category variability such as position, angle, and size of the stimuli that resulted in amplitude modification. Moreover, the difference in K2.1 response amplitude to fearful, happy, neutral, and no form provides strong evidence for an emotional effect. Future research should consider implementing other non-face emotional stimuli to address the question of stimulus specificity.

Taken together, we were able to detect responses to emotional faces in early V1 processing *via* nonlinear multifocal VEPs over the occipital cortex, implying that there is differential early visual processing of emotional faces with the M pathway connections of V1. In particular, we found that fearful faces at 70% temporal contrast produce a greater M pathway nonlinearity than do happy or neutral faces. Further exploration of putative feedback and response gain modulation models will be needed to fully explain the VEP differences observed.

## Data Availability Statement

The datasets generated for this study are available on request to the corresponding author.

## Ethics Statement

The studies involving human participants were reviewed and approved by Swinburne Human Research Ethics Committee, Swinburne University of Technology. The patients/participants provided their written informed consent to participate in this study.

## Author Contributions

EM created the experimental design, performed testing and data collection, analyzed the data, and wrote the manuscript. DC contributed to stimulus creation and manuscript editing. Both authors contributed equally to interpreting the results.

## Conflict of Interest

The authors declare that the research was conducted in the absence of any commercial or financial relationships that could be construed as a potential conflict of interest.

## Footnotes

^1^http://www.VPixx.com
